# Case Report: Delayed Lung Transplantation With Intraoperative ECMO Support for Herbicide Intoxication-Related Irreversible Pulmonary Fibrosis: Strategy and Outcome

**DOI:** 10.3389/fsurg.2021.754816

**Published:** 2021-11-26

**Authors:** Guohui Jiao, Xiangnan Li, Bo Wu, Hang Yang, Guoqing Zhang, Zheng Ding, Gaofeng Zhao, Jingyu Chen

**Affiliations:** ^1^Wuxi Lung Transplant Center, Wuxi People's Hospital Affiliated to Nanjing Medical University, Wuxi, China; ^2^Department of Thoracic Surgery, The First Affiliated Hospital of Zhengzhou University, Zhengzhou, China

**Keywords:** herbicide, pulmonary fibrosis, lung transplantation, ECMO, hemopurification

## Abstract

**Background:** Lung transplantation is recognized as the only therapeutic option for patients who develop irreversible pulmonary fibrosis after herbicide intoxication.

**Methods:** We have collected and presented clinical course and outcome of four patients who received lung transplantation due to paraquat and diquat intoxication from 2018 to 2021. Another patient who received initial lung transplantation due to paraquat intoxication and re-transplantation due to chronic lung allograft dysfunction in 2019, was further reported. Patients were admitted in lung transplantation centers, including the 1^st^ affiliated hospital of Zhengzhou University and Wuxi Lung transplantation center. Previous reported cases from Europe, Canada and China were also summarized as benchmark.

**Results:** During the period from the year of 2018 to 2021, there have been four patients in China, who received lung transplantation due to herbicide intoxication. Median age of the four patients was 37 (IQR 34.5, 39.75) years old. Median time from intoxication to lung transplantation was 27.5 (IQR 27, 30.5) days. Bilateral lung transplantation was performed in three patients, while one single lung transplantation was performed in an urgent listed patient. Extracorporeal Membrane Oxygenation (ECMO) and hemopurification support were used in all patients (100%). Details of the cases with follow-ups were further presented and analyzed.

**Conclusions:** Late timing of bilateral lung transplantation can be performed successfully for pulmonary fibrosis after paraquat or diquat intoxication. The survival of patients with complex perioperative conditions can be achieved with a multidisciplinary team to manage the irreversible effects of intoxication.

## Introduction

The compound 1,1′-dimethyl-4,4′-bipyridinium dichloride, known as paraquat, is a widely used, highly toxic contact herbicide that has been banned in many countries. As no antidotes exist, paraquat causes severe and potentially fatal intoxication (1). Other analogs, such as diquat, demonstrates similar pathological changes to the lungs (2). Paraquat can be taken up by the tissues and cleared by the kidney. High levels of paraquat could still be detected as late as 9 weeks after ingestion (3). The lungs have been recognized as the main target organs that are injured by the active accumulation of paraquat, which results in irreversible pulmonary fibrosis. Respiratory failure has been recognized as the main cause of death in the late phase of paraquat intoxication. The current treatments for patients with paraquat poisoning are mostly empirical and supportive, such as early gastric lavage to prevent absorption, emesis induction to promote excretion, laxative administration, and hemoperfusion for detoxification, together with the administration of antioxidants, immunosuppressive agents and even mesenchymal stem cells (4–8).

Since the lung transplantation technique was established, reports have shown that patients suffering from paraquat intoxication could receive lung transplantation as a salvage therapy after exhausting all existing therapeutic regimens (1, 9). Progressive lung damage has been demonstrated without any doubt. Time for considering lung transplantation referral and evaluation, as well as the perioperative support strategy, were discussed in published cases. Here, we summarized the cases performed in China, as well as in the European and North American centers (1968–2017), serving as a useful reference for clinicians in the future.

## Patients and Methods

### Study Design and Data Collection

We retrospectively analyzed the medical records of patients from the 1^st^ affiliated hospital of Zhengzhou University (Case 1–4), Wuxi Lung transplantation center and Beijing Chaoyang Hospital (Case 5), submitted to lung transplantation due to herbicide-induced-lung fibrosis or re-transplantation, between 2018 and 2021. Information collected from all patients included demographic data, surgical details, and follow-up data. Another case was admitted and treated in the 1^st^ hospital of Guangzhou Medical University, while detailed data has been reported by Jiang et al. (10). Early cases reported with detailed data from Edinburgh, Toronto and Geneva were also summarized and benchmarked to demonstrate the transition of therapeutic strategy on transplantation timing and perioperative support. Summary of cases are shown in Table 1. Continuous data with normal distributions are presented as median with interquartile ranges.

**Table 1:** Case summary.

	**Case 1**	**Case 2**	**Case 3**	**Case 4**	**Case 5**	**Case 6** **(Guangzhou,2019)**	**Case 7** **(Edinburgh,1968)**	**Case 8** **(Edinburgh,1973)**	**Case 9** **(Toronto, 1985)**	**Case 10** **(Geneva, 1997)**
Year	2018	2020	2020	2021	2014/2019^*^	2017	1968	1973	1982	1995
Gender	Male	Male	Male	Male	Female	Female	Male	Male	Male	Male
Age	45	38	36	30	24/29	26	15	18	31	17
Poison volume/concentration	60 ml/20%	50 ml/20%	80 ml Diquat	60 ml/20%	50 ml/20%	20 ml/20%	1 mouthful	1 mouthful	Clothes drenched Chronic exposure	Chronic exposure
Poison concentration (ug/ml) at 1st admission	Urine, 229.2	Urine, 148.6	Urine,139.7	Urine, 126.4	Urine, 248.96	NR	Blood, 0.4		Blood, 0.26	Lung, 0.134
Gastric Lavage	Yes	Yes	Yes	Yes	Yes	Yes	NR	NR	Yes	NR
Hemoperfusion/ dialysis	Yes	Yes	Yes	Yes	Yes	Yes	Yes	Yes	Yes	Yes
P/F	150	97	50	124	50	85	70	66	69	69
From poisoning to ECMO bridging (days)	–	–	26	–	44	–	–	–	–	–
From poisoning to LT (days)	38	27	27	28	56	58	6	10	32, 51	44
Type of LT	Bilateral	Bilateral	Single	Bilateral	Bilateral	Bilateral	Single	Single	Single sequential Right then Left	Single
ECMO mode	V-V	V-A	V-A^*^+ V-AV	V-V	V-V	V-V^*^+V-A	No	No	V-V	No
Post-LT hemodialysis	Yes	Yes	No	No	No		No	NR	Yes	Yes
**Post-LT complication**										
Infection	Yes	Yes	Yes	Yes	Yes	Yes	NR	NR	Yes	NR
Thrombogenesis	Yes	No	No	No	No	NR	NR	NR	No	NR
Bronchial stenosis /Fistula	Yes	No	No	Yes	No	NR	NR	NR	trachea-innominate artery fistula	Bronchopleural fistula
Acute pancreatitis	Yes	No	No	No	No	NR	NR	NR	No	No
Cardiac failure	No	No	Yes	No	No	NR	NR	NR	No	No
Neuromyopathy	No	No	No	No	No	NR	NR	NR	Yes	Yes
Survival status	>3 years	>7 months	Death on sepsis	>7 months	Retransplantation on 5th year, survived > 20 months	>1 year	Death on respiratory failure	Death on respiratory failure	Death of massive cerebral infarction	>1 year

*NR, not reported*;

* *ECMO was used pre-operatively as bridging to lung transplantation*.

### Ethics

The Institutional Ethics Committees of all centers involved in this study approved the procedures, including verbal consent procedures and data collection. Written informed consent was obtained from the patients and next of kin. The transplanted organs were obtained from volunteer donations, and the next of kin voluntarily provided written informed consent. No lungs were obtained from executed prisoners. The Institutional Ethics Committees of the Organ Procurement Organization approved the donation procedures. Donor lungs were allocated through the China Organ Transplant Response System. The National Transplant Medical Review Board (Chinese Lung Transplantation Society and Transplantation Data Management & Quality Control Center) approved and registered the donors' and recipients' data.

### Perioperative Assessment and Perioperative Management

Preoperative investigations included lab tests, lung function evaluation, functional assessment of other vital organs, and anesthetic evaluation. Preoperative chest X-ray or computed tomography (CT) examinations did not reveal any pulmonary infections or other pulmonary diseases among the donors with PaO_2_/FiO_2_ (P/F) >300 mmHg.

Patients received gastric lavage, antioxidants, immunosuppressive agents, hemopurification as conservative therapy. However, bilateral lung fibrosis still progressed as shown in chest imaging (Figures 1.1–4A). Patients had progressively worsening dyspnea and low P/F ratio. Continuous renal replacement therapy, hemoperfusion or hemodialysis was performed due to acute kidney injury in most cases per admitted centers' protocols. Although patients could be stabilized on liver and renal function, while paraquat was not detected in urine, patients could not be weaned from intubation and pulmonary fibrosis progressed. Lung transplantation was thus performed for these patients.

**Figure 1:**
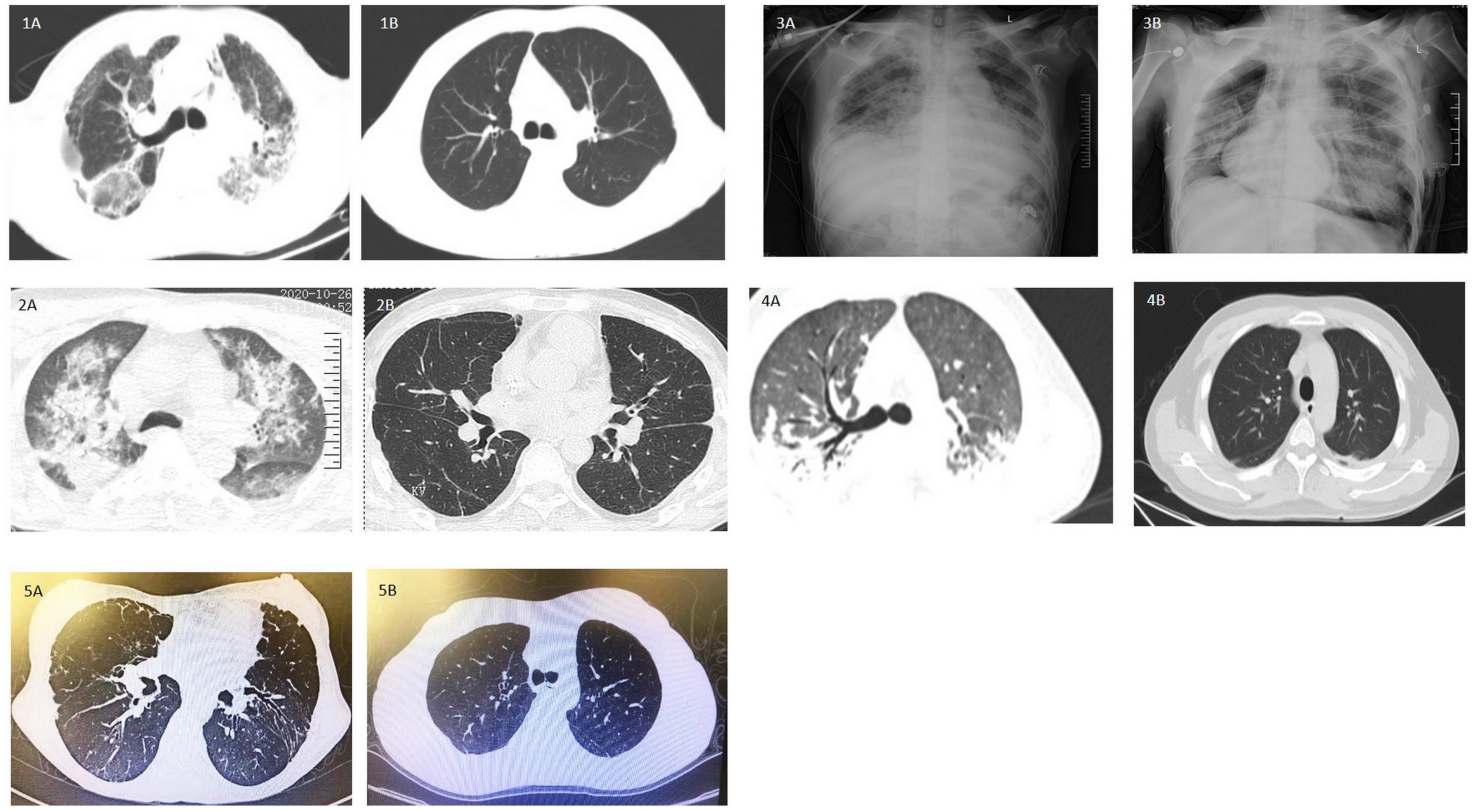
CT images of five patients pre-lung transplantation and post-lung transplantation. **(1A−4A)** Progression of bilateral lung fibrosis with consolidation were shown in case 1–4. **(1B, 2B, 4B, 5B)** Post-lung transplantation CT manifestation without significant abnormality. **(3B)** Chest imaging showed significant lung effusion and edema in Case 3. **(5A)** Case 5 suffered deteriorated graft function after 5 years of transplantation and chronic lung allograft dysfunction was suspected.

Postoperatively, all patients were admitted in ICU. The immunosuppressive drugs administered included mycophenolate mofetil, tacrolimus, and prednisone. All patients received prophylactic antimicrobial and antiviral medications to prevent bacterial, fungal, and viral infections. Postoperative blood examinations, chest radiography, and bronchoscopy were performed per routine institutional protocol.

## Results and Case Presentation

**Figure 2:**
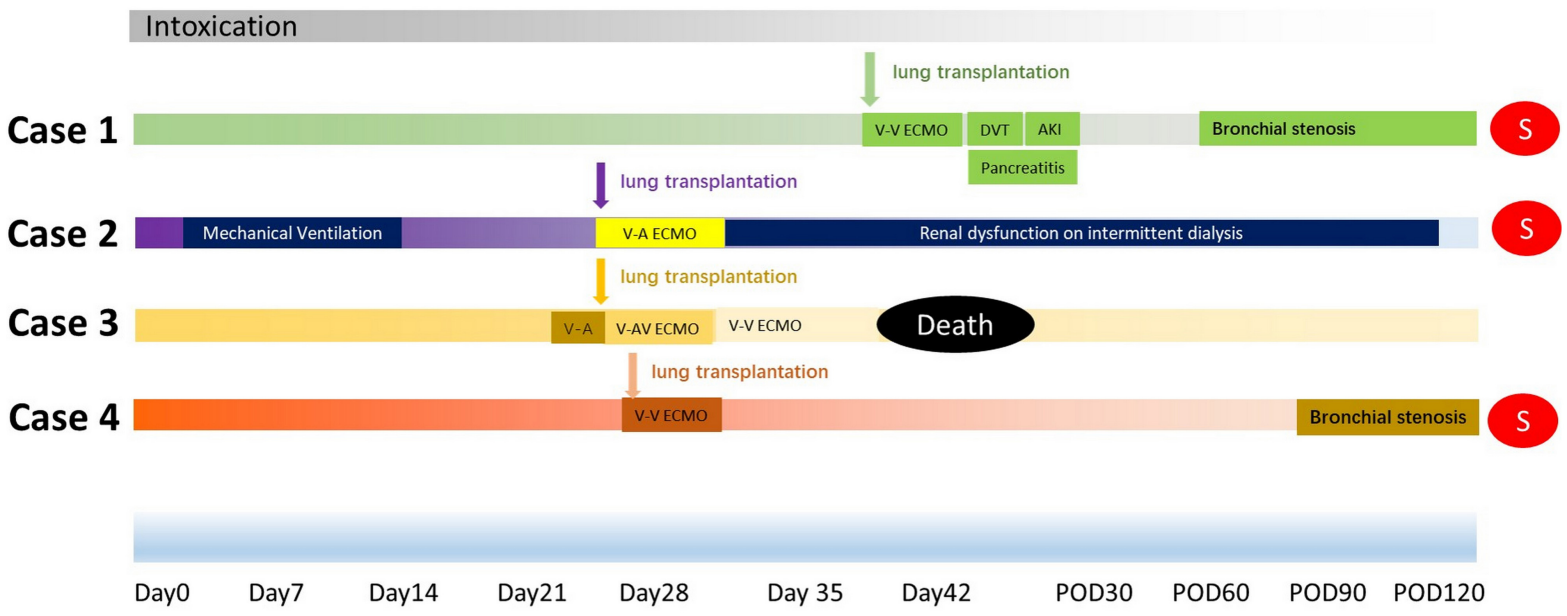
Illustration of clinical course and perioperative events in Case 1–4. Pre-lung transplantation and intraoperative mechanical ventilation support, ECMO support modes and duration were illustrated. Post-lung transplantation events were labeled with outcome up to the follow-up date. S, survival; DVT, deep vein thrombosis; AKI, acute kidney injury; POD, post-operation day.

We have collected and reported the details of four cases who received lung transplantation for the 1^st^ time from 2018 to 2021 (Case1–4). The median age of the patients (Case1–4) was 37 (IQR 34.5, 39.75) years old. Median time from intoxication to lung transplantation was 27.5 (IQR 27, 30.5) days. Three patients (75%) received bilateral lung transplantation and ECMO was used for intraoperative support in all four patients (100%). Various ECMO support modes, including venovenous(V-V) (Case 1, 4), venoarterial(V-A) (Case 2, 3) as well as hybrid mode V-AV (Case 3) were all feasible to support patients. Hemopurification, including hemodialysis and hemoperfusion, were used in all patients (100%). The clinical course of Case 1–4 are presented in Figure 2. Baseline lab tests and perioperative characteristics were collected and presented in Supplementary Table 1. In addition, we presented the long-term follow-up of another case (Case 5) who received initial lung transplantation due to paraquat intoxication and re-transplantation due to chronic lung allograft dysfunction.

Multiple perioperative complications occurred in these patients. Case 1 developed deep vein thrombosis on postoperative day 2 (POD2) and an inferior vena cava filter was implanted. Acute pancreatitis was diagnosed on POD14 after initiating oral feeding. After receiving conservative therapy as per institutional protocol, patient's status was stabilized and recovered on POD39 and discharged. During the routine follow-up, on POD61, bilateral bronchial stenosis in the proximal termino-terminal bronchial anastomosis was observed. Cryosurgery under bronchoscopy was performed on POD 64, POD67 and POD101 under general anesthesia. Case 4 also experienced bronchial stenosis and was treated by cryosurgery under bronchoscopy. These patients survived to date with improved respiratory symptoms and no obvious lung abnormalities on CT images (Figures 1.1B, 4B). In Case 2, episodes of infection occurred and fully resolved 1 month after lung transplantation. Hemodialysis was continued up to 4 months after surgery. Upon follow-up, patient has fully recovered renal function and pulmonary imaging was normal (Figure 1.2B). All these three cases have been regularly followed up in lung transplantation clinics. They survived and returned to work.

Emergent V-A ECMO support for acute respiratory distress syndrome as bridging to lung transplantation was performed in Case 3. Urgent single lung transplantation was performed under V-AV ECMO to improve oxygenation. Mode of ECMO support was transited to V-V ECMO support post-lung transplantation. Cardiac arrhythmia and grade 3 primary graft dysfunction occurred sequentially and could not be wean-off ECMO (Figure 1.3B). Patient died on POD12 due to sepsis caused by multi-drug resistant organisms' infection.

From the perspective of long-term outcome, Case 5 had bilateral lung transplantation in the year of 2014 and survived up to 5 years post-lung transplantation. The clinical course of her 1^st^ lung transplantation has been reported previously in detail (11). Patient had progressively worsening dyspnea and headache from the 4^th^ year after surgery. Significant elevation of pulmonary artery pressure (40 mmHg) was detected. Pseudomonas aeruginosa was cultured from sputum. Lung function showed FEV1 0.48 L and FVC 1.19L with deteriorating chest imaging of chronic lung allograft dysfunction (Figure 1.5A). Bilateral lung transplantation was performed and postoperative recovery period was uneventful (Figure 1.5B). Follow-up lung function showed FEV1 1.9 L, FVC 2.29 L.

## Discussion

Paraquat was introduced to the market as an herbicide in 1962 (12). Diquat is recognized as the second widely used herbicide in the world (13). Over the last few decades, numerous paraquat intoxication cases have been reported, mainly caused by accidental or voluntary ingestion or extensive skin contamination. Much fewer cases of diquat intoxication have been reported in the literature than those of paraquat poisoning, thus, clinicians are still warranted to give attention to its high mortality rate. Due to the abundant polyamine transport systems of type I and II alveolar cells and Clara cells, the lungs are believed to be the main target organ of paraquat poisoning. Paraquat that has accumulated in the lungs and muscle remains even after it is no longer present in blood or urine (14). Mechanism of diquat toxicity is also related to oxidative stress and cell death, aggravating the damage of lungs and kidneys (15).

Respiratory symptoms after intoxication vary from no manifestations to severe shortness of breath, leading to death within as short as 24 h but typically within 2–4 weeks. Treatment strategy for herbicide intoxication, includes gastric lavage, antioxidant therapy, hemoperfusion and hemodialysis as well as immunosuppressive agents. All our patients received the treatments listed above, bilateral pulmonary fibrosis still progressed to the irreversible stage and acute respiratory distress syndrome manifested. All the cases performed in China after 2017 had access to more life support strategies, such as hemopurification and ECMO, which could be used to support organ function and bridge to transplant.

Early cases reported in Europe and Toronto had high mortality rate before 1997 (3, 9, 16, 17). Apart from lack of life-support modalities, single lung transplantation was performed in most cases. All those patients received transplantation within 1 month after intoxication, paraquat could be detected in the blood and death was unavoidable in short term. Toronto team tried secondary contralateral lung transplantation after the initial graft failed. Having been the pioneer of lung transplantation in the world, Toronto team has performed prolonged ECMO support and trachea-innominate artery fistula was well-detected and managed. Death still occurred due to massive cerebral infarction, which is also recognized as the most fatal complication of ECMO.

The case reported by Geneva team after 1997 achieved long-term survival success (3, 17). Single lung transplantation was performed, however, the time for transplantation was delayed to 44^th^ day, beyond 1 month after intoxication. Toxic neuromyopathy still existed while hemodialysis continued post-lung transplantation. The patient recovered and survived for over 1 year as reported. This case was the first to demonstrate that late timing of lung transplantation could benefit patients on long-term survival. In our report, patients received lung transplantation after 1 month from intoxication. Further, bilateral lung transplantation was performed in most cases. Only Case 3 received single lung transplantation in an urgent manner and did not survive. Thus, late timing of bilateral lung trans-plantation might be critical for saving these patients' lives.

**Figure 3:**
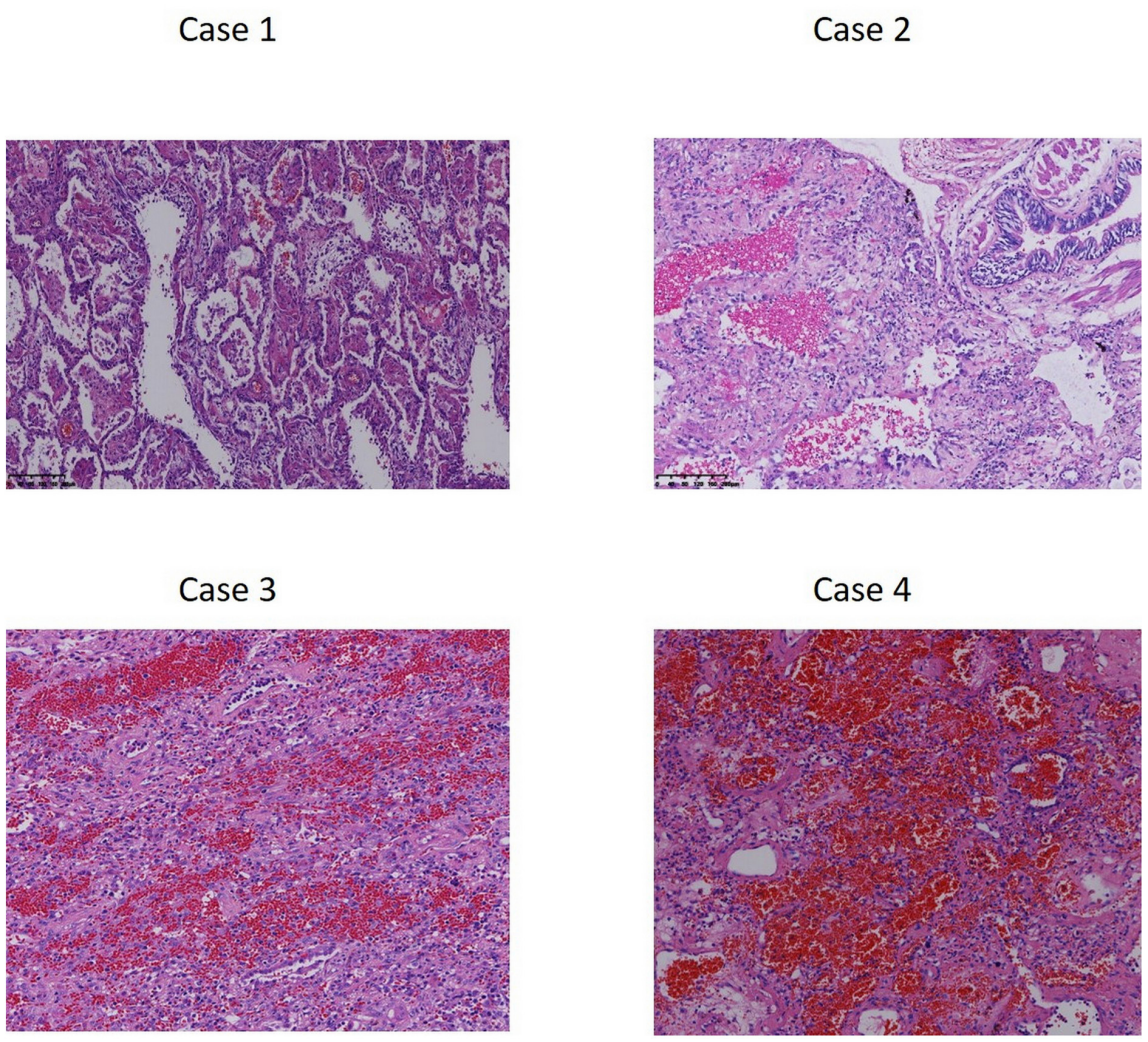
Pathology examination of explanted lung tissue after herbicide intoxication. Massive fibroblastic tissue obliterated the lung architecture, with extensive effusions, capillary congestion, inflammatory cell infiltration, alveolar edema, and hyaline membrane formation, could be observed with varied extent of severity in Case 1–4.

Like in other end-stage pulmonary fibrosis patients, ECMO is crucial for intraoperative lung transplantation support and bridging to urgent lung transplantation. While combining with the treatment of hemopurification, more herbicide could be cleared, thus ensuring the long-term benefit for post-lung transplantation survival. In addition, immunosuppressive drugs are also effective for transplantation as well as the intoxication. Poison was not detected in the explanted lungs of our cases. Pathology examination showed extensive effusions, inflammation and congestion in all the intoxicated lungs (Figure 3).

Long-term follow-up for these patients is mandated to ensure life quality as they received transplantation in their middle ages. Some of the patients may have unstable emotion before intoxication or even experienced suicidal tendencies. After we evaluated their psychological status thoroughly, we determined to list them as lung transplantation candidates. Close cooperation between doctors and patients is of key importance for maintenance of long-term graft function. The Case 5 patient is a famous singer in China. After re-transplantation, doctors encouraged the patient to continue vocal training and the patient went back to performing on stage. All the patients who were transplanted and survived cherished their lives, continued treatment and followed up regularly in outpatient clinics.

Our experience of successfully treating these patients highlights the efficacy of multi-disciplinary approaches in such complicated scenarios, which could guide clinical practice and inspire the management of these patients who wish to live. Patients after herbicide intoxication showed rapid progression of irreversible pulmonary fibrosis and even acute respiratory distress syndrome. When considering lung transplantation referral timing and strategy, bilateral lung transplantation after 1 month of intoxication with hemopurification and ECMO support will be crucial for long-term survival. These patients warrant physical and psychological rehabilitation during follow-up. Potential intrinsic correlations between poisonous effects and post-operative complications must be explored to further improve quality of life.

## Data Availability Statement

The original contributions presented in the study are included in the article/Supplementary Material, further inquiries can be directed to the corresponding author/s.

## Ethics Statement

The studies involving human participants were reviewed and approved by Wuxi People's Hospital affiliated to Nanjing Medical University and the First Affiliated Hospital of Zhengzhou University. The patients/participants provided their written informed consent to participate in this study. Written informed consent was obtained from the individual(s) for the publication of any potentially identifiable images or data included in this article.

## Author Contributions

JC and GJ: conceptualization. XL and GZhao: resources. BW, HY, GZhang, and ZD: data curation. GJ and XL: writing—original draft preparation. JC and GZhao: writing—review and editing. All authors contributed to the article and approved the submitted version.

## Conflict of Interest

The authors declare that the research was conducted in the absence of any commercial or financial relationships that could be construed as a potential conflict of interest.

## Publisher's Note

All claims expressed in this article are solely those of the authors and do not necessarily represent those of their affiliated organizations, or those of the publisher, the editors and the reviewers. Any product that may be evaluated in this article, or claim that may be made by its manufacturer, is not guaranteed or endorsed by the publisher.

## References

[1] Cooke NJ Flenley DC Matthew H. Paraquat poisoning. Serial studies of lung function. Q J Med. (1973) 42:683–92. 4606886

[2] Wang D Zhang G Zhang W Luo J Zhu L Hu J. Successful extracorporeal membrane oxygenation support for severe acute diquat and glyphosate poisoning: a case report. Medicine. (2019) 98:e14414. 10.1097/MD.0000000000014414 30732194 PMC6380784

[3] Licker M Schweizer A Hohn L Morel DR Spiliopoulos A. Single lung transplantation for adult respiratory distress syndrome after paraquat poisoning. Thorax. (1998) 53:620–1. 10.1136/thx.53.7.620 9797765 PMC1745280

[4] Meredith TJ Vale JA. Treatment of paraquat poisoning in man: methods to prevent absorption. Human Toxicol. (1987) 6:49–55. 10.1177/096032718700600108 3546086

[5] Lavergne V Nolin TD Hoffman RS Roberts D Gosselin S Goldfarb DS. The EXTRIP (EXtracorporeal TReatments In Poisoning) workgroup: guideline methodology. Clin Toxicol. (2012) 50:403–13. 10.3109/15563650.2012.683436 22578059

[6] Lin JL Lin-Tan DT Chen KH Huang WH Hsu CW Hsu HH. Improved survival in severe paraquat poisoning with repeated pulse therapy of cyclo-phosphamide and steroids. Int Care Med. (2011) 37:1006–13. 10.1007/s00134-010-2127-7 21327593

[7] Gawarammana IB Buckley NA. Medical management of paraquat ingestion. Brit J Clin Pharmacol. (2011) 72:745–57. 10.1111/j.1365-2125.2011.04026.x 21615775 PMC3243009

[8] Tsai HL Chang JW Yang HW Chen CW Yang CC Yang AH. Amelioration of paraquat-induced pulmonary injury by mesenchymal stem cells. Cell Transpl. (2013) 22:1667–1681. 10.3727/096368912X657765 23051186

[9] Matthew H Logan A Woodruff MF Heard B. Paraquat poisoning–lung transplantation. Brit Med J. (1968) 3:759–63. 10.1136/bmj.3.5621.759 4877735 PMC1989570

[10] Jiang WZ Chen YQ Zhang YL Zhang TT Liu YM Xu X. Lung transplantation in patients with paraquat poisoning: a case report and literature review. Zhonghua Lao Dong Wei Sheng Zhi Ye Bing Za Zhi. (2019) 37:292–6. 10.3760/cma.j.issn.1001-9391.2019.04.013 31177699

[11] Tang X Sun B He H Li H Hu B Qiu Z. Successful extracorporeal membrane oxygenation therapy as a bridge to sequential bilateral lung transplantation for a patient after severe paraquat poisoning. Clin Toxicol. (2015) 3:908–13. 10.3109/15563650.2015.1082183 26314316

[12] Dinis-Oliveira RJ Duarte JA Sanchez-Navarro A Remiao F Bastos ML Carvalho F. Paraquat poisonings: mechanisms of lung toxicity, clinical features, and treatment. Crit Rev Toxicol. (2008) 38:13–71. 10.1080/10408440701669959 18161502

[13] Fortenberry GZ Beckman J Schwartz A Prado JB Graham LS Higgins S Lackovic M. Magnitude and characteristics of acute paraquat- and diquat-related illnesses in the US: 1998–2013. Environ Res. (2016) 146:191–9. 10.1016/j.envres.2016.01.003 26775000 PMC4920265

[14] Saunders NR Alpert HM Cooper JD. Sequential bilateral lung transplantation for paraquat poisoning. A case report. The toronto lung transplant group. J Thorac Cardiovasc Surg. (1985) 89:734–42. 10.1016/S0022-5223(19)38729-X 3887042

[15] Jones GM Vale JA. Mechanisms of toxicity, clinical features, and management of diquat poisoning: a review. J Toxicol Clin Toxicol. (2000) 38:123–8 10.1081/CLT-100100926 10778908

[16] Kamholz S Veith FJ Mollenkopf F Montefusco C Nehlsen-Cannarella S Kaleya R. Single lung transplantation in paraquat intoxication. N Y State J Med. (1984) 84:82–4. 6366653

[17] Walder B Bründler MA Spiliopoulos A Romand JA. Successful single-lung transplantation after paraquat intoxication. Transplantation. (1997) 64:789–91. 10.1097/00007890-199709150-00026 9311725

