# Supplementary material for: Case Report: Delayed Lung Transplantation With Intraoperative ECMO Support for Herbicide Intoxication-Related Irreversible Pulmonary Fibrosis: Strategy and Outcome
Source: Front Surg. 2021 Nov 26;8:754816. doi: 10.3389/fsurg.2021.754816 (PMC8660696; doi:10.3389/fsurg.2021.754816)
Supplement: Supplementary file 1 [file Table_1.DOCX]

Supplementary Table1 Baseline clinical manifestation, lab results pre-lung transplantation and perioperative characteristics

|  | **Case 1** | **Case 2** | **Case 3** | **Case 4** |
| --- | --- | --- | --- | --- |
| **Main symptom before LT evaluation** | Severe dyspnea | Dyspnea, palpitation | Chest pain, dyspnea | Chest pain, dyspnea |
| **WBC (*10^9^/L)** | 7.03 | 12.13 | 19.1 | 7.37 |
| **Hemoglobin (g/L)** | 91.2 | 74 | 83 | 73.5 |
| **ALT (U/L)** | 19 | 20 | 39 | 24 |
| **TBIL (μmol/L)** | 6.6 | 18.2 | 18.7 | 12.7 |
| **Creatinine (μmol/L)** | 73 | 429 | 98 | 171 |
| **Prothrombin time (s)** | 14.1 | 13.9 | 15.3 | 17.5 |
| **D-dimer (mg/L)** | 2.05 | 5.60 | 3.13 | 0.9 |
| **Donor type** | DCD | DBD | DBD | DCD |
| **Intraoperative blood loss (mL)** | 500 | 1000 | 800 | 1200 |
| **Post-LT FEV1 (L)** | 1.88 | 1.16 | - | ND |
| **Post-LT FVC (L)** | 2.79 | 2.24 | - | ND |

DBD, donation after brain death; DCD, donation after cardiac death; ND, not detected.; LT, lung transplantation.
